# Apoptotic effect of thymoquinone on OVCAR3 cells via the P53 and CASP3 activation

**DOI:** 10.1590/acb399224

**Published:** 2024-11-08

**Authors:** Özge Karaosmanoğlu, Zeynep Kamalak, İlhan Özdemir, Şamil Öztürk, Mehmet Cudi Tuncer

**Affiliations:** 1 Acibadem Maslak Hospital, IVF Clinic – Istanbul – Turkey.; 2 Ağrı İbrahim Çeçen University – Faculty of Medicine – Department of Gynecology and Obstetrics – Ağrı – Turkey.; 3 Atatürk University – Faculty of Medicine – Department of Gynecology and Obstetrics – Erzurum – Turkey.; 4 Çanakkale Onsekiz Mart University – Vocational School of Health Services – Çanakkale – Turkey.; 5 Dicle University – Faculty of Medicine – Department of Anatomy – Diyarbakir – Turkey.

**Keywords:** Cell Cycle, Apoptosis, Antineoplastic Agents, Ovarian Neoplasms, Cell Line

## Abstract

**Purpose:**

The limitations in cancer treatment and the inadequacy of classical methods have made it necessary to discover therapeutics in cancer treatment. The cytotoxicity of thymoquinone, which has quite different properties in terms of biological activities, in ovarian cancer cells, and the changes in the expression levels of apoptotic genes (p53/caspase-3 (casp-3)) were investigated.

**Methods:**

In the study, thymoquinone (5, 50, 100, 250 and 500 µM and 24, 48, 72 hours) were applied to ovarian adenocarcinoma cancer cell line (OVCAR3), at different concentrations. Cytotoxic effect of thymoquinone on OVCAR-3 cells were analyzed by MTT method, and apoptotic and pro-apoptotic gene expression levels (p53, Casp-3) of thymoquinone in cancer cells were analyzed by quantitative real-time polymerase chain reaction.

**Results:**

Thymoquinone, whose effect has been revealed in many types of cancer, was shown to significantly reduce the viability of OVCAR3 cancer cells depending on the dose and time (*p* < 0.05). It was also determined that Casp-3 and p53 gene expressions increased in OVCAR3 cells.

**Conclusions:**

Considering the in-vitro cytotoxic activity and apoptotic gene expressions of thymoquinone, an important treatment agent, since it is a promising agent for the future of cancer treatment, more comprehensive studies may pave the way for its clinical use.

## Introduction

Due to its asymptomatic course, late diagnosis, and high potential for recurrence, ovarian cancer is the third most common gynecological malignancy with the highest mortality rate among cancers^1^^,^ 2. When diagnosed, it is usually accompanied by intraperitoneal metastasis. Current data show that ovarian cancer contributes to recurrence because it develops resistance to traditional chemotherapeutics^3^^,^ 4. Despite of the abundance of different subtypes of ovarian cancer, its exact etiology has not been defined yet, and, since these cancers tend to occur mostly in advanced stages, it is important to well define the molecular mechanisms underlying its development.

Nowadays, many plant-based treatments are applied as complementary treatments against cancer. One of the substances used in this complementary treatment is thymoquinone in black cumin^5^. This plant (*Nigella sativa*) is used in traditional medicine for the treatment of many diseases. It has been demonstrated that thymoquinone (TQ) and dithymoquinone, the main active components of volatile fatty acids contained in *Nigella sativa* seeds, have antineoplastic, antibacterial, antifungal, antihelminthic, immunostimulatory, anti-inflammatory, antioxidant, and hypoglycemic effects^6^. Despite the antineoplastic, antibacterial, immunostimulatory, anti-inflammatory, and antioxidant properties of TQ, its mode of action has not been fully elucidated to date. However, it is reported that TQ induces apoptosis in breast and gastric cancer^7^.

Apoptosis is a highly conserved and important form of cell death for cell development, suppression of oxenogenesis, and host attack. Cancerous or infected cells are eliminated by apoptosis. The defect in this basic mechanism is the main cause of tumor development and progression^8^. This mechanism is mediated by many proteins and genes. Caspase and Bcl-2 genes play important roles in the regulation and maintenance of apoptosis. The amplification of the apoptotic signal and the proteolytic processing of many cellular target molecules are achieved by the activation of caspases^9^. The balance of proteins that initiate and stop apoptosis is the main factor determining the fate of cells.

The damage in DNA caused by different factors stops the cell cycle for repair or apoptosis to occur^10^. The greater the DNA damage, the more effective the repair activities must be so that the balance affecting cell viability is not disrupted. When DNA is damaged, the cell either takes itself from the G1 phase to the S phase or pauses by switching from the G2 to the M phase. Studies have shown that the p53 gene plays a central role in cell cycle control and cell survival. When a cell is damaged, it either repairs the cell or drives it to apoptosis depending on the extent of the damage^11^.

In ovarian adenocarcinoma cancer cell line (OVCAR3) cells treated with TQ, it was observed that activated p53 and the mechanism that stops the cell cycle and induces apoptosis were activated. In addition, it was determined that the caspase cascade was activated with mitochondrial transformations. The anticancer properties of TQ, one of the important compounds whose studies on cancer are ongoing in the current literature, were investigated. Moreover, the properties of an agent that can be used as an alternative treatment to traditional chemotherapy and that can reduce the effectiveness of chemotherapy agents were investigated.

## Methods

### Cell culture

In this study, frozen vial OVCAR3; ATCC Catalog No. HTB-161 cells obtained from ATCC, were used. After the cells were thawed in a water bath, they were grown in a medium containing 10% fetal calf serum and RPMI 1,640 medium. Cell growth was monitored daily under a microscope. Subculture of monolayer cells in cell culture dishes was performed using trypsin-versen medium.

### Cytotoxicity

The stock solutions of TQ (Aldrich Co.) used in the study were prepared using ultrapure ethanol (Merck, United States of America). One hundred mM TQ stock solution was prepared and stored in aliquots at -20ºC. The vehicle in 96-well culture dishes was reduced to 0.1%. To determine TQ IC_50_ doses, OVCAR3 was seeded in 96-well culture dishes with multipipetting at 3,000–5,000 cells per well. After one night, TQ was applied at different concentrations with serial dilutions in the dose ranges of 5, 50, 100, 250, 500 µM and incubated for 24, 48 and 72 hours. For the MTT test, cells were separated into TQ and vehicle control groups and multiplied in 6-well plates. After incubation, cells were subjected to viability analysis. For MTT, 5 mg/mL yellow tetrazolium (3-(4, 5-dimethylthiazolyl-2)-2,5-diphenyltetrazolium bromide) test solution was prepared, and 20 µL was added to each well. After incubation for 4 hours, the medium in the wells was cleared, and 200 µL dimethyl sulfoxide (DMSO) (Merk, United States of America) was added and kept in the dark for 2–4 hours. The slides were read with a microplate reader (Thermo Scientific, United States of America) at 492–650-nm wavelengths. The control group was taken as a reference to determine 100% viability value, and the viability ratio was calculated comparatively. IC_50_ results for the OVCAR3 cell line were calculated using the Statistical Package for the Social Sciences (SPSS) 20.0 program.

### Cell morphology

Nuclear morphology changes and apoptotic structures caused by control and TQ agents in OVCAR3 cell line were determined with NucBlue Live ReadyProbes Reagent (Thermo Scientific, United States of America) specific stain. In this context, OVCAR3 were seeded in 24-well plates as 5 × 10^4^ cells/well. The vehicle and TQ IC50 were added to these wells the next day. Then, staining was performed as direct live cell staining according to the kit protocol, and cells were incubated for 30 minutes. Afterwards, plates were photographed through Thermo EVOS FL Imaging System using brightfield mode and fluorescence mode using DAPI filter at 20X objective magnification.

### Real-time quantitative polymerase chain reaction

In the study, expression levels of P53, CASP3 genes in control and treatment groups of OVCAR-3 were analyzed by real-time quantitative polymerase chain reaction (RT-qPCR). The primers used are given below in 5’-3’ order:

P53: F: CACGAGCGCTGCTCAGATAGC, R: ACAGGCACAAACACGCACAAA, CASP3: F: GGTATTGAGACAGACAGTGG, R: CATGGGATCTGTTTCTTTGC, β-Actin: F: CCTCTGAACCCTAAGGCCAAC, R: TGCCACAGGATTCCATACCC, GAPDH; F: CGGAGTCAACGGATTTGGTCGTAT, R: GCCTTCTCCATGGTGGTGAAGAC

cDNAs obtained from isolated RNAs were used in gene expression studies. cDNA samples were performed in RT-qPCR in accordance with the Power Sybeer Green qPCR MasterMix (thermo, United States of America) kit protocol. Applied Biosystems QuantStudio device was used to amplify cDNAs. For the qRT-PCR reaction to take place, some steps were performed:

- Enzyme activation: 95ºC-10 min;
- Denaturation: 95ºC-15 s;
- Primer annealing-Chain extension: 60ºC-1 min;
- Melting curve: 95ºC-15 s, 60ºC-1 min, 95ºC-15 s.

The peaks obtained from the amplification were used to determine Ct gene expressions, and the results were calculated by the 2-∆∆Ct method. As endogenous control, β-actin and glyceraldehyde 3-phosphate dehydrogenase (GAPDH) mRNA expressions were used as calibration and correction factors.

### Protein-protein interaction analysis

Protein-protein interaction (PPI) data were retrieved from the STRING database. The STRING database provides descriptions of PPIs, as well as confidence intervals for data scores. A confidence score greater than or equal to 0.4 was chosen to construct the interaction network of proteins with target genes.

### Enrichment analysis

Data on the functional annotation of genes and the canonical pathways associated with the strong connections established with these proteins were obtained using the ShinyGO 0.80 program.

### Gene ontologies functional enrichment analysis

Three types of gene ontologies (GO) were performed on possible target genes: cellular component (CC), biological process (BP), and molecular function (MF). The SRplot bioinformatics program was used to evaluate these data.

### Statistical analysis

The difference between the cell viability values determined by the MTT test and the gene expressions obtained by RT-qPCR was determined by one-way analysis of variance (ANOVA), the groups in which the averages fell were determined by the Tukey’s HSD test, and the comparisons between the two groups were determined by the t test or Mann-Whitney’s U test. For all statistical evaluations, the SPSS 20 program and *p* ≤ 0.05 value were used.

## Results

### Cytotoxicity

**Figure 1:**
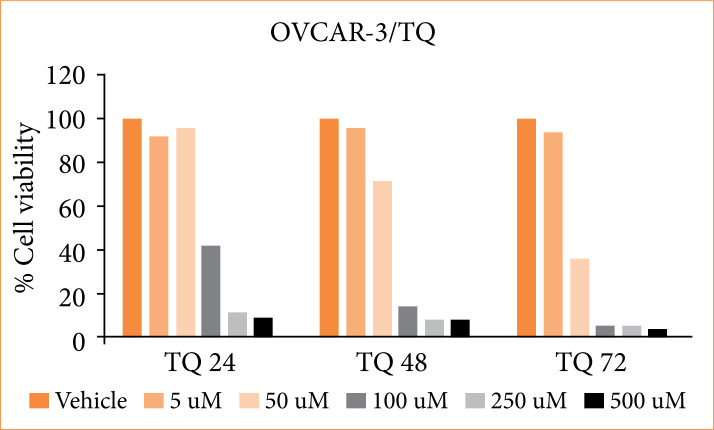
Effect of thymoquinone applied to OVCAR3 cells at six different concentrations for 24, 48 and 72 hours on % cell viability.

Source: Elaborated by the authors.

The % cell viability and IC50 value of OVCAR3 cells obtained with MTT test in our study are shown in Fig. 1. After TQ treatment, it was seen that cell viability in OVCAR3 cell line decreased below 50% after 24 hours at the concentration of 50 µM. Twenty-four-hour IC50 was found to be 90.42 µM. After 48 hours of TQ treatment, cell viability decreased below 50% after the concentration of 50 µM, and 48-hour IC_50_ was found to be 65.4 µM. After 72 hours of TQ treatment, cell viability decreased below 50% after the concentration of 50 µM, and 72-hour IC50 was found to be 40.2 µM. One-way ANOVA Tukey’s HSD test was made between vehicle group and different concentrations of TQ. In statistical comparisons, significance was calculated according to p ≤ 0.05. The decrease in cell viability after 50 µM (24, 48 and 72 hours) TQ treatment showed statistical significance (Fig. 1).

### NucBlue staining

**Figure 2:**
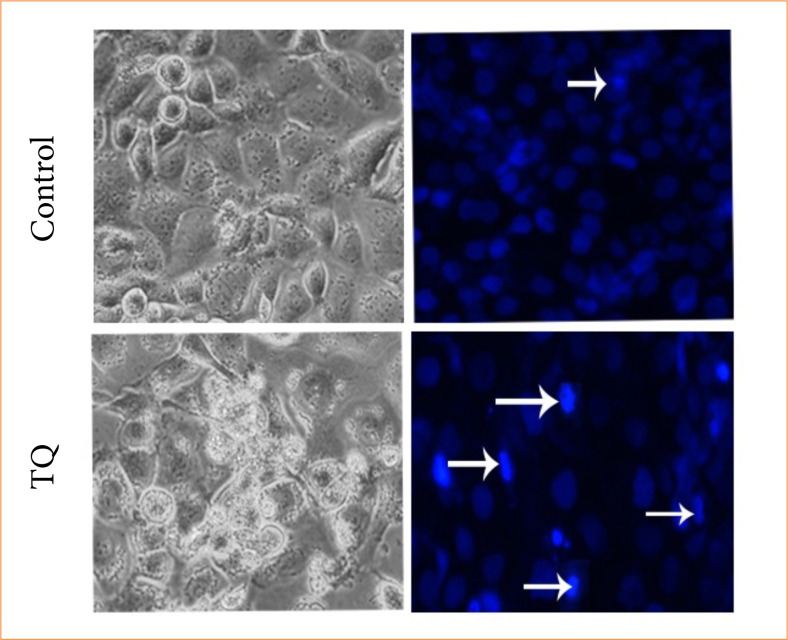
Cell morphology, nuclear structure, and apoptotic body formation in OVCAR3 ovarian carcinoma cell populations treated with vehicle control and thymoquinone (TQ) IC50 for 48 hours.

Source: Elaborated by the authors.

NucBlue staining was performed on all samples after TQ treatment. It was determined that cell death increased in the cells after TQ treatment depending on the application dose. It was determined that cell proliferation was also suppressed in NucBlue staining and therefore the number of cells decreased. The nuclear fragmentations occurring in the cell nucleus caused the cells to take on a bright appearance. This indicates that apoptosis has occurred (Fig. 2).

### Quantitative real-time polymerase chain reaction analysis of apoptotic markers

**Figure 3:**
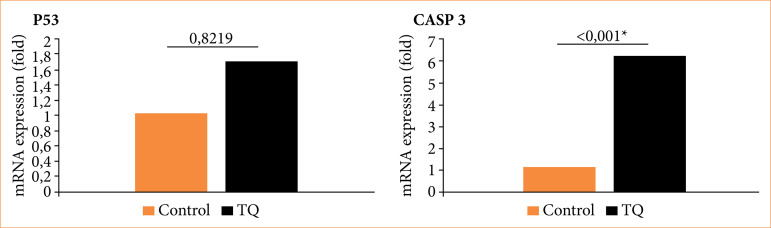
P53 and CASP3 mRNA expression ratio of thymoquinone (TQ)-treated OVCAR3 cells and the control group (*p* < 0.0001 = *).

Source: Elaborated by the authors.

In RT-qPCR analyses, TQ IC_50_ was applied to the experimental groups determined by MTT analyses and NucBlue staining for 48 hours. RT-qPCR was applied to a total of nine samples in all groups; and proapoptotic P53 and apoptotic Caspase-3 gene expressions were used by normalizing them to the internal control β-actin expression of the same sample. P53, CASP3, and β-actin gene expressions were determined at detectable levels, and amplification curves were created. As a result of the calculations, an increase in P53 and CASP3 was observed in the TQ group compared to the 48-hour control group. However, the increase in p53 gene expression did not contain statistical significance. It was determined that CASP3 gene expression (RQ = 0.5) was expressed at the highest level with an 80% increase in the TQ group. CASP3 gene expression in the TQ group showed a statistically significant difference compared to the control group (*p* < 0.0001) (Fig. 3).

### Protein-protein interaction analysis

**Figure 4:**
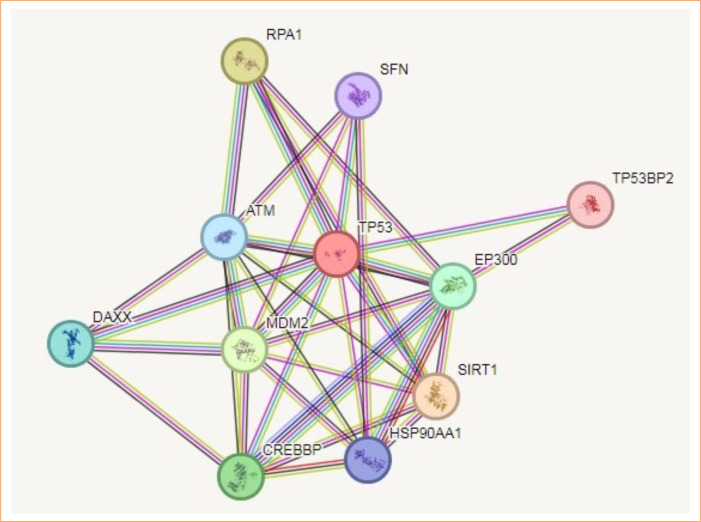
Protein-protein interaction and interaction between various genes of ovarian cancer.

Source: Elaborated by the authors.

Predictions from STRING analysis were used to depict protein interactions. The visualization showed 11 nodes and 35 edges (Fig. 4). Based on nodal degree, the following genes were identified as the top 10 central genes: RPA1, ATM, DAXX, CREBBP, HSP90AA1, EP300, TP53BP2, SFN, MDM2, and SIRT1. These targets were hypothesized to be the primary targets in ovarian cancer of TQ.

### KEGG pathway enrichment analysis

**Figure 5:**
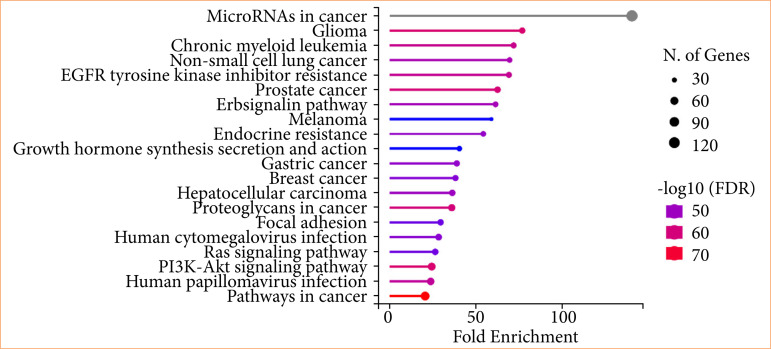
Enrichment analysis for the 153 common compound targets.

Source: Elaborated by the authors.

KEGG pathway enrichment analysis of target genes was performed with Shiny 0.80 program. The findings showed that 153 genes were involved in the enrichment process, and 90 pathways were cancer-related, exhibiting a significant correlation with target genes (*p* < 0.05). Basically, MicorRNAs in cancer, glioma, chronic myeloid leukemia, non-small cell lung cancer, EGFR tyrosine kinase inhibitör resistance, prostate cancer, ErbB signaling pathway, melanoma, endocrine resistance, and growth hormone synthesis the top 10 pathways that occur in diabetic complications are shown in Fig. 5.

### Gene ontologies functional enrichment analysis

**Figure 6:**
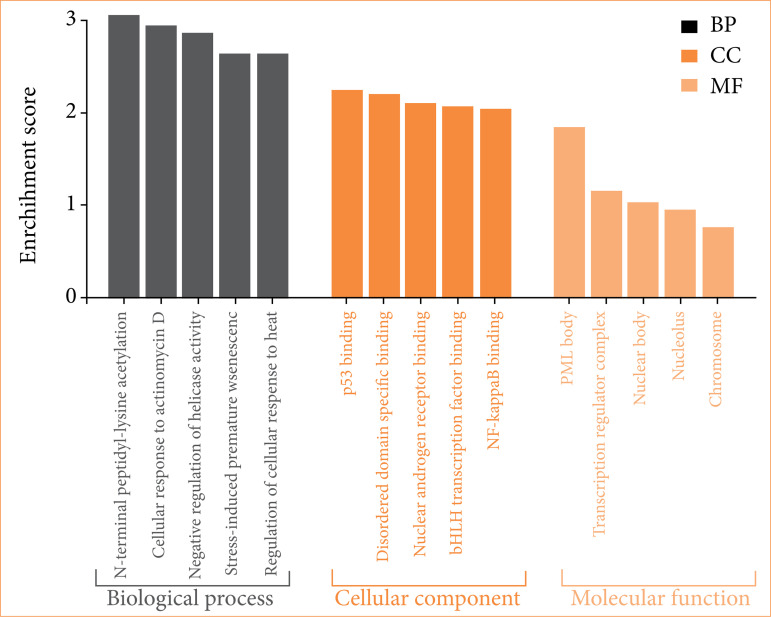
Gene ontologies (biological process, cellular component and molecular function) analysis.

BP: biological process; CC: cellular component; MF: molecular function. Source: Elaborated by the authors.

Analysis findings showed only important functions (Fig. 6). Target genes were found to be involved in various cellular components in the BP category, such as N-terminal peptidyl-lysine acetylation, cellular response to actinomycin D etc. (Fig. 6). In terms of cellular components, there were promyelocytic leukaemia (PML) body, transcription regulator complex. It was found that the MF category exhibited roles such as p53 binding, disordered domain specific binding (Fig. 6).

## Discussion

Although there has been a war against cancer worldwide for years, its morbidity and mortality have not decreased^12^. Every year, enormous amounts of money are spent on cancer research, and various methods have been developed for its treatment, but a decrease in the incidence of the disease cannot be achieved. The main reason for this is that most of the drugs used in cancer treatment usually cause negative side effects, such as liver dysfunction, ischemic heart disease, fatigue, hypertension and vomiting, which further hinder the treatment process and eventually cause cancer progression^13^^,^ 14. In addition, cancer cells becoming resistant to chemotherapy drugs is not a desired situation in terms of the success of the treatment^15^. Therefore, it is an important step to develop treatment strategies that are easily accessible and low cost, with high efficiency and minimal side effects for the management of cancer treatment, which is a deadly disease, and to bring them to the clinic^12^. Preventive and therapeutic strategies in the fight against cancer, such as phytochemicals and chemotherapeutic agents, are being discovered. It is equally important to determine and investigate the effects of these substances. Phytochemicals such as polyphenolics, terpenes, phenolics, alkaloids and sulfur compounds are obtained from a wide variety of fruits, vegetables and plants that we consume in our daily lives and have tremendous anticancer activities^16^. Moreover, these natural agents can modulate growth factors, various cell receptors, chemokines and cytokines, transcription factors and molecular oncogenic targets naturally maintained within the cell. In this way, cancer cells can be made targets for chemotherapeutic drugs, the side effects of drugs used in treatment can be reduced, the quality of life of patients can be increased and, most importantly, cancer patients can extend their lives for a long time^17^^,^ 18.

Flavones constitute one of the subgroups of flavonoids, which are the most important bioactive compounds in the group of secondary metabolites. Flavones have anti-inflammatory, antioxidant, anticarcinogenic, antiproliferative, antiangiogenic, biochemical, and pharmacological activities and do not have toxic effects. The therapeutic potential of flavones makes these compounds valuable targets for drug design.

In this context, it is important to focus on the use of flavones in cancer prevention and treatment^19^. Although many chemotherapy drugs are very effective treatment agents, their use is limited because they have serious side effects, trigger vital organ failures and cause resistance to the drug in cancer cells. Natural compounds with anticancer activity can be put into action at this point and an important problem can be solved. In this respect, we showed how effective TQ is on the viability of cancer cells with the MTT method in our study findings. We also found that it has anticancer activity on OVCAR3 cells. TQ can be a promising treatment agent because it shows the highest cytotoxic effect according to MTT analysis.

Plants have taken the first place among living beings in the discovery of active substances in cancer treatment. The fact that the newly discovered compounds have very few or negligible side effects and their strong fighting abilities against cancer cells have made these substances alternatives to traditional treatments. There are many natural compounds such as TQ, curcumin, rosmarinic acid, and alpha lipoic acid, and these compounds hold great promise for the production of new cytotoxic drugs^20^^,^ 21. The main goal is to reduce or completely eliminate the side effects of existing chemotherapy. Antioxidant treatments are sometimes used for this. The side effects of free radicals are reduced or eliminated with plant compounds. The effectiveness of antioxidants in protecting tissues from oxidative stress can vary, and this variability depends on the antioxidant’s biopharmaceutical properties, type, concentration reached in the area of effect and the nature of oxidative stress^21^.

The mortality rate of cancer is increasing every year and causes the death of hundreds of thousands of people. Unfortunately, standard treatments such as chemotherapy drugs, radiotherapy or immunotherapy, which is becoming more and more important every day, are only successful in a portion of patients due to the high heterogeneity of the disease. In gene therapy or treatments developed for molecular targets, a single gene or a product of that gene or the signaling pathway responsible for the expression of that gene is targeted to eliminate a specific group of cells in the tumor. Other genetically different cell variations with carcinogenic properties can easily escape treatment and circulate as cells with the capacity to form tumors in the surrounding area. Therefore, in cancers, such a feature often eventually reveals drug resistance^22^^–^ 24.

The cause of these poor results is due to genomic instability and abnormal activation of genes involved in the tight protection of DNA. Platinum-based chemotherapies have been developed to overcome this problem. In these treatments, in order to eliminate the failure of cancer treatments, drugs are applied by reshaping, preventing repair of DNA damage and increasing genotoxic sensitivity that triggers cell death^25^^,^ 26. The ability of TQ to control the growth rate of OVCAR3 cells and its inhibition was exhibited with a significant decrease depending on the 48-hour period and dose. The results obtained were found to be close to certain IC_50_ doses of TQ with pancreatic cancer cells^27^^,^ 28. We obtained the highest cytotoxic effect of TQ alone on ovarian cancer cells after 48 hours with a dose of 62.9 µM. Another study reported that the results of A549 cells driven to apoptosis were increased with TQ treatment. The effect of TQ on increasing the Bax/Bcl-2 ratio has been revealed, and it has been shown to induce apoptosis and increase p53 expression^29^. Moreover, it has been shown that TQ, in combination with spironolactone, causes apoptosis by activating caspase 3/7 on other cancer cells^30^^,^ 31.

The reason for the differences between the IC_50_ values of TQ in the OVCAR3 cell line in our study can be explained by the fact that, although the MTT test has been used in cancer research for about 30 years, different results are obtained in each measurement. Some cell lines create resistant responses, especially in absorbance measurement, causing different results to be obtained in the same measurements^32^^–^ 34. It is very rare for IC_50_ values to be consistent. A given chemical compound rarely produces different IC_50_ values in a cancer cell line. He et al.^35^ reported that the reason for this problem are the formulas used by the manufacturers and laboratories where the products are produced. It has been reported that sometimes, even for the same laboratory product, IC_50_ values can differ between different repetitions of experiments, even when MTT results are performed by different researchers. The reason for these inconsistencies can be explained by the variability in the control wells when calculating IC_50_ values^35^. When we look at the study results, a parallelism was found between TQ-induced apoptosis and cell viability obtained as a result of MTT. We determined that thymoquinone activates P53 and Caspase-3 pathways while driving OVCAR3 cells to apoptosis.

## Conclusion

There are still hopeless treatment regimens for some subtypes of ovarian cancer. At this point, TQ may be an alternative to platinum-based drugs traditionally used in the treatment of ovarian cancer. Moreover, it has advantages such as lower toxicity and higher efficiency. It is recommended that TQ be studied in more detail in different types of cancer, developed with animal models and finally carried to the clinic, and more detailed pharmaceutical and efficacy studies are carried out.

## Data Availability

The data will be available upon request.
